# Temporary Urbanisms as Policy Alternatives to Enhance Health and Well-Being in the Post-Pandemic City

**DOI:** 10.1007/s40572-021-00314-8

**Published:** 2021-04-20

**Authors:** Lauren Andres, John R. Bryson, Paul Moawad

**Affiliations:** 1 Bartlett School of Planning, University College London, London, UK; 2 Birmingham Business School, University of Birmingham, Birmingham, UK

**Keywords:** Temporary urbanism, Temporary uses, Health, Built environment, Post-pandemic city, Adaptability, Health-led temporary urbanism

## Abstract

**Purpose of Review:**

While there has been extensive discussion on the various forms of temporary uses in urban settings, little is known on the ways in which temporary and health urbanisms connect. Now, a turning point has been reached regarding the interactions between health and the built environment and the contributions made by urban planning and other built environment disciplines. In the context of the post-pandemic city, there is a need to develop a health-led temporary urbanism agenda than can be implemented in various settings both in the Global South and North.

**Recent Findings:**

Health-led temporary urbanism requires a reinterrogation of current models of urban development including designing multifunctional spaces in urban environments that provide sites for temporary urbanism-related activities. A healthy city is an adaptable city and one that provides opportunities for citizen-led interventions intended to enhance well-being by blending the temporary with the permanent and the planned with the improvised.

**Summary:**

Health-led temporary urbanism contributes to the call for more trans- and inter-disciplinary discussions allowing to more thoroughly link urban planning and development with health.

## Introduction

Health and cities are intrinsically linked. The development of planning as a profession and as a policy field in the C19^th^ was incentivised by the need to address significant public and environmental health issues [1]. Since then, planning practice and research has focused on the interactions between health and urban development in the context of the regulation of land use. However, more trans- and inter-disciplinary discussions are required. [2] recently considered evidence-informed planning for healthy liveable cities and stressed that health researchers need to undertake policy-relevant research and understand policy-making processes to influence city planning. Similarly, Pineo [3•] argued that while the fields of health and sustainability have been increasingly connected to those of urban design and the development of healthy built environments [3•, 4], there still remain many built environment practitioners who do not consider health and sustainability objectives to be part of their work [5, 6].

Over the last decade, urban planners have been called on to address health and well-being inequalities and to improve living conditions for diverse groups, especially the most vulnerable [7–9]. This has been a priority at local, national, and international levels driven by the World Health Organization and United Nations [3•]. A growing academic orthodoxy has emerged arguing that health and urban well-being (including mental well-being), and the wider social and environmental challenges cities are facing, need to be addressed at a micro-level and require place-based approaches combined with macro-level interventions. This has been reinforced by the COVID-19 pandemic which has highlighted the need to recentre this debate further. This is where ‘temporary urbanism’ [10, 11] appears to provide important insights into micro-level and place-based approaches which connect planning and health. Temporary urbanism includes ‘processes, practices and policies of and for spatial adaptability, which allow the activation of a space in perceived need of transformation, thus leading to paths of change through a trajectory of transformation’ ([11•], p.2). The ‘temporary’ is one pathway towards enhancing urban health and well-being.

A turning point has been reached regarding the interactions between health and cities and the contributions made by the built environment disciplines (e.g. urban planning, design, and architecture). This can be explained by two factors. First, cities and urban spaces must be able to cope with change and adaptability by facilitating processes of smooth transformation in line with very diverse, rapid, or slower disruptions, of various natures and strengths. This has led to an increase in temporary uses of urban land/buildings. Tactical and bottom-up interventions over the last decade have been driven as part of a planned strategy based on a step-by-step process of redevelopment of buildings, high streets, and public spaces by local authorities, landowners, and developers [10, 11]. Second, global shocks, including the current pandemic, and those which may follow [12] coupled with the climate change crisis and its significant environmental and social upheavals continue to ripple across urban environments. Both factors highlight that cities must be able to adapt, transform, and bounce towards other status and forms of stability. There is an important paradox here—urban areas have rigidity built into them based on the historic accumulation of fixed capital investments, but they also must be adaptable. The resilience of places and people is key here. This highlights the ways in which ‘temporary urbanism’ connects to ‘healthy urbanism’ [3•], encompassing prerogatives including (mental) well-being, physical exercise, and overall quality of life while being inclusive of the need to limit transmission of respiratory tract infection pathogens. To date, there has been limited discussion on the ways in which the temporary and health urbanism connect; this is the focus of this paper.

To do so, this paper will review and discuss insights from existing literatures and research exploring pre- and post-pandemic (COVID-19) cities focussing on micro/neighbourhood interventions to draw recommendations and wider lessons for both practitioners, policy-makers, and researchers. Importantly though this paper is not about the COVID-19 pandemic, COVID-19 is considered as a planning inflection moment in which existing approaches to urban development must be re-evaluated. This re-evaluation has just commenced, but considerable attention has previously been given to temporary adaptations and adjustments, and this will be enforced further. To do so, we start by deconstructing the concept of temporary urbanism and how it has spread as a widely used approach to characterise temporary uses of urban spaces in very diverse contexts. We then move to unwrap the ways in which temporary and healthy urbanisms connect through a range of short illustrations. This leads us to sketch out the directions towards health-led temporary urbanism and draw recommendations for future areas of practice, research, and policy. The discussion mobilises insights from several recent research projects conducted between 2016 and 2020 in the UK, Brazil, South and East Africa, and Lebanon.

### The Facets of Temporary Urbanisms

Temporary adaptations to land and buildings have been a feature of cities for several decades. Empty, vacant, and sometimes derelict lands or buildings are occupied in response to a diversity of drivers and needs [13, 14]. Until recently no common terminology existed to conceptually assemble the diversity of uses, projects, and occupations and durations. Terms including ‘DIY urbanisms’ and ‘pop-ups’ [15], ‘tactical urbanisms’ [16], ‘insurgent place making’ [17], ‘differential spaces’ [18], and ‘weak planning’ [19] emerged in the literature (see Harris [20••] for a detailed review). In policy and practice, the term ‘meanwhile’ has been used widely, typically in the 2020 London Resilience Strategy [21]. The concept of ‘temporary urbanism’ was initially developed by Madanipour [22] and further elaborated by Andres et al. [23], Andres and Kraftl [11•], and Andres and Zhang [10•]. Agreement has emerged around a shared concept based on understanding the evolution of urban environments as an outcome of a process that combines planned long-term interventions with more temporary practices. Temporary urbanism returns adaptability to the centre of the urban place-making process.

Temporary urbanism offers a shared language to consider the diversity of temporary uses while enabling a dialogue to emerge between experiences and practices developed in very different urban contexts, both in the Global South and North [23]. Adaptability involves two main features, creative adaptability based on innovative forms of urban living and allowing/encouraging experimentation and testing of new ideas, led by artists, local businesses, communities [10•], and adaptability as more informal and unplanned practices are linked to everyday coping for the most vulnerable communities. This is an important point, that is developed in the next section, which underpins narratives that sit at the intersection between temporary urbanism and health.

Temporary urbanism has three main origins. First, as noted by Andres and Kraftl [11•], ‘cities result from a constant process of construction and reconstruction, based on redundancy and re-use’ [24, 25]. As a result, ‘the built environment is never fully stable and completed; by essence the “unfinished” is part of the urban condition’ [26]. Urban obsolescence opens new pathways for temporary solutions to emerge, while more permanent land use adaptations are developed and assessed. Adaptability is thus also part of the urban condition [11•]. Urban planners have been reluctant to acknowledge the contribution that temporary urbanism makes to urban diversity, well-being, and health. Temporary approaches to urban place-making have tended to be driven by artists, architects, and urban designers rather than planners or city councils. Second, temporary urbanism is linked to creative urban place-making, urban imaginaries, and forms of improvisations which see beyond vacancy and regard emptiness and un-use as an opportunity to propose alternative but temporary occupations. This is where the prefiguration of temporary urbanism connects with artistic squats and illegal cultural occupations. Finally, temporary urbanism relates to informality and insurgency particularly in cities of the Global South where a significant urban cohort live and cope every day through alternative practices and occupations of urban space ([27, 28, 23]). Scholars and policy-makers began to engage with the temporary in urban settings three decades ago. Nevertheless, adaptable and flexible use of space has characterised cities for a long time ([23]). To date, three types of temporary urbanism can be identified and summarised as follows [10•].

The first is *bottom-up temporary urbanism*. It relates to unplanned and more informal uses and occupations driven and implemented outside any formal planning and/or regulated frameworks. This form of temporary urbanism is embedded within a context of transition and has an important duration element: it constitutes an in-between and uncertain status between a stage ‘A’ (see, e.g. Andres [19] or Moawad [29]) which led to development of a temporary form of urbanism to a stage ‘B’, which is often unknown highlighting the importance of flexibility. The second type is *top-down temporary urbanism*; it embraces the latest and most contemporary urban trend where temporary uses and projects are no longer perceived as blockages for redevelopment but on the contrary are considered as a mechanism to leverage change and activate early-stage transformation, in formal settings [10•]. Such forms of temporary urbanism have been used widely in the UK (see, e.g. Bishop and Williams [30••] and Bishop [31, 32]), Germany [33], France [34], Switzerland [35], China [36], or Chile [37–39], embedded within formal reimaginings of cities and neighbourhoods and wider strategies of urban transformation. The third type relates to *hybrid temporary urbanism* emphasising the variable nature of temporary urbanism and its complexity. This is closely related to severe and sudden disruptions and the need for immediate and rapid adaptation; ‘hybridity is here reflected in the processes of bricolage amongst key stakeholders who construct and develop temporary uses, meaning that boundaries between regulatory powers and power to take back ownership of spaces (specifically open/public spaces) are blurred’ (Andres, 2020, p.3). These uses are important in the context of sudden disruptions, for example, the COVID-19 pandemic.

These different forms of temporary urbanisms are connected through three core concepts: adaptability, activation, and trajectory [11•]. These concepts also connect temporary with healthy urbanism. Adaptability engages with the economic and ecological resilience literature ([40]) and includes processes of activation of use, of behaviour, and of change. Adaptability also opens possibilities for alternative path creation, with the potential to transform place and people enabling alternative citizen-led pathways to be identified and enacted producing better outcomes for citizens and places. Activation relates to ‘value’ understood as not only an economic and financial construct but also as a social and cultural construct [41]. The notion of value highlights the intrinsic nature of temporary urbanisms as responses to socio-economic or human crisis, contexts of transition, and more importantly major or minor dysfunctions in the urban (development) system. It also stresses their roles in generating alternative trajectories of transformation, with diverse spatial, economic, and social repercussions, and forms of valorisation. Finally, it sits within a process of transitioning within a wider trajectory of transformation, as a form of testing what values and outcomes can be generated, using temporary interventions that are anticipatory and strategic particularly towards raising the perception of an area and, of course, correlatively land values. In other contexts, this transitioning process refers to situations of daily coping and/or waiting denoting a process of using makeshift skills to meet basic needs.

The economic and social benefits and challenges of temporary urbanism have been widely discussed in the literature, but the connections to health have only been explored indirectly, for example, by considering the consequence of temporary allotments on communities (see Bishop and Williams [30••]). The COVID-19 pandemic and its wider consequences act as a turning point that have highlighted the impact that temporary urbanism has on cities and people. The next section explores the ways in which temporary urbanism and health are intrinsically linked.

### Temporary Urbanisms and Health in Urban Settings

The connection between temporary urbanism and health can be uncovered in three ways (see Table 1), and each, as we will explore in the following section, has wider policy implications: first in tackling overall well-being and liveability within the context of urban spaces; second in ensuring everyday coping for vulnerable communities including access to basic needs; and third, in constituting rapid solutions in the context of crisis, while also being considered as unhealthy urban features. These are considered in turn.

**Table 1:** The three facets of temporary urbanisms and health in urban settings and their policy implications

Case studies	Forms of temporary urbanisms	Connection to health	Wider implications for health-led temporary urbanisms
1. Temporary gardening-type activities, temporary playgrounds, temporary ‘community’ spaces	*Top-down temporary urbanism* Aimed at leveraging change and activate early-stage transformation, in formal settings; embedded within formal reimaginings of cities and neighbourhoods	*Well-being and liveability*: healthy lifestyle behaviours, tailored to address health inequalities, liveability, social integration and inclusion, and promoting physical exercise	Intersectional approaches positioning people’s need first and applying temporary urbanism approaches to generate wider health benefits.
2. Unplanned and unregulated uses of space and practices	*Bottom-up temporary urbanism*. Unplanned and more informal uses and occupations; sit outside of any formal planning and/or regulated frameworks; context of transition and waiting; everyday coping and improvisation	*Everyday coping and access to basic needs:* provision of shelter and alternative coping routes through the informal economy and food systems; off-grid access to water or electricity; linked to dire (and unhealthy) living conditions	Addressing exclusion through an acceptation of permanent-impermanence; recognising the importance of experiential learning and the role of creative temporary urbanism in meetings basic health needs
3 COVID-19/crisis-related temporary adaptations and adjustments to respond to specific needs and concerns	*Hybrid temporary urbanism* Linked to severe and sudden disruptions and the need for immediate and rapid adaptation; process of bricolage amongst diverse stakeholders, role of creativity and experimentation	*Crisis, health, and temporariness:* focused on resilience and need for adaptability for well-being, liveability, and for health precautionary measures; stigmatisation of everyday coping and informal forms of temporary urbanism leading to a reduction of community resilience and increasing vulnerabilities	Design urban environments to facilitate rapid but temporary alterations in the relationship between people and urban space. Promote individual and household well-being along with social connections while improving social care networks. Design a changeable, permutable, and adaptable healthy city

#### Well-Being and Liveability

Resting upon the social determinants of health [42], the urban social and physical environments are inextricably linked with societal outcomes and prevailing health inequalities [43]. An individual’s interaction with the social environment plays a key role in the development of healthy lifestyle behaviours over the life course [44]. Derelict sites, buildings, lands, but also transport corridors or parking spaces are elements of a city’s physical structure which through temporary interventions can shift from being ‘anti-spaces’ to becoming liveable spaces contributing to overall well-being and social integration. Over the last decade, temporary urbanism has been widely used as a powerful tool to support healthy lifestyles, well-being, and physical activity while addressing concerns about improved liveability and inclusion of people into wider urban transformations. We illustrate this with the example of London and Sao Paulo.

**Fig. 1:**
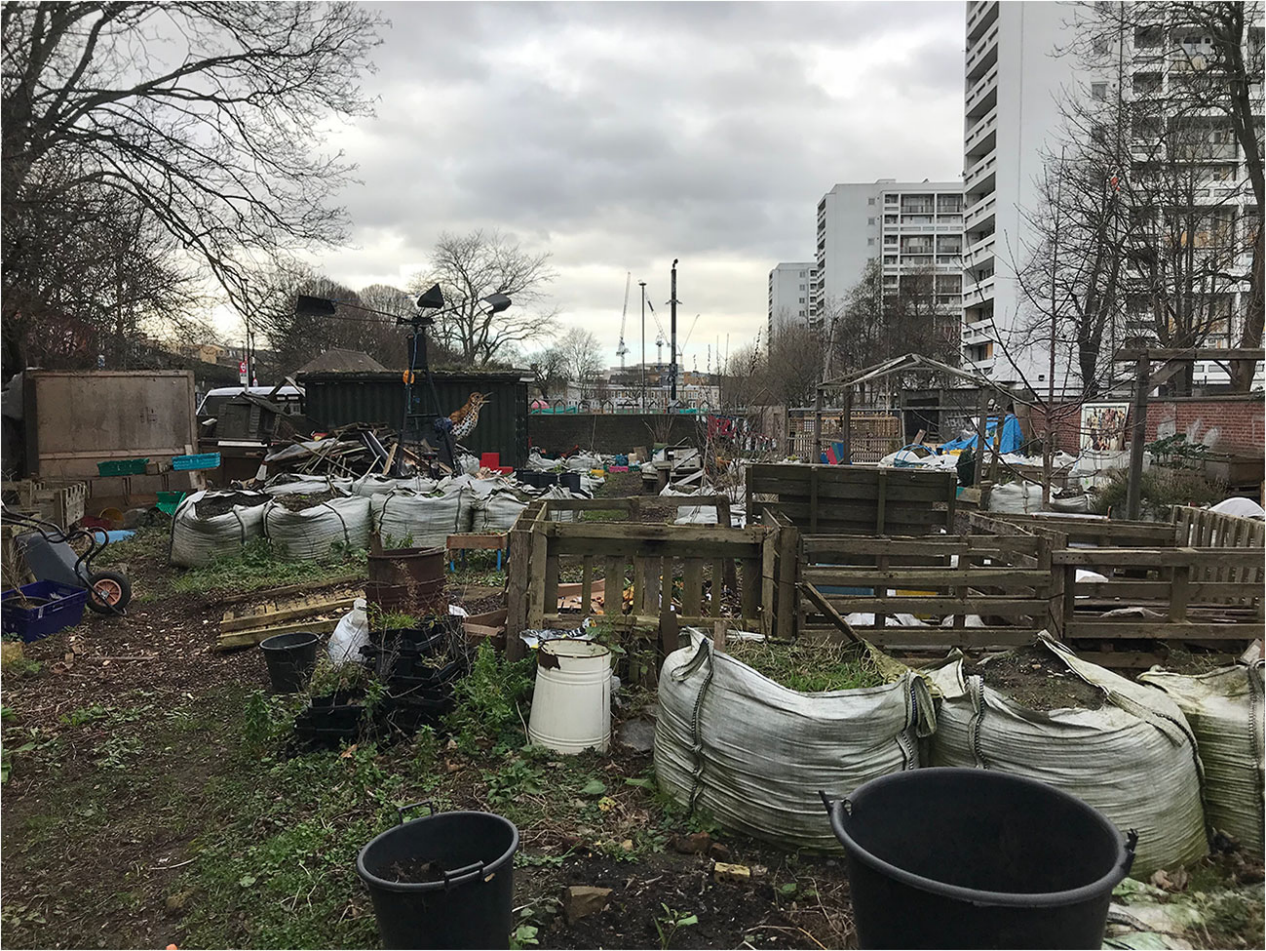
The Loughborough Farm project (Andres, 2018)

In London, the spread of top-bottom strategies for temporary urbanisms has been led by key decision-makers including landowners, local authorities, and developers (see, for instance, [45]; London [46, 47]). These strategies rest upon the use of temporary interventions as non-permanent solutions to leverage future trajectories of urban development within ‘in-between’ situations. A significant proportion of temporary urban interventions have been motivated by highly complex property markets in the context of rapidly rising land values and housing shortages. It also occurs in a city riven with stark socio-economic inequalities. Formal temporary urban interventions have aimed to both adapt to local property markets while also serving the needs of communities. Temporary uses were widely used during the King's Cross St. Pancras redevelopment project through temporary gardening initiatives (Skip Garden project) and the King’s Cross Pond Club, a public art project based on the creation of a temporary micro-ecological environment with a natural swimming pond at its centre. This testifies to the fact that grand designs should not exclude the temporary but should incorporate opportunities for temporary urbanism. In Loughborough Junction, manifold smaller-scale initiatives have aimed to animate under used spaces. It has included using small empty spaces, unfit for redevelopment (e.g. railway arches to back gardens) for community gardening projects (see Fig. 1), constructed as moveable installations (including topsoil contained in bulk bags that can be transported from one place to another), to support food growing which is then either used in the temporary community coffee shop or sold on a weekly basis. The Loughborough Farm project, run by volunteers in collaboration with neighbourhood forums, tenants and resident associations, youth centres, general practitioner practices, local artists, and businesses, is formed around several temporary gardens ‘designed to bring people together to improve well-being and decrease isolation’ while also making the borough ‘greener and healthier’ [48].

**Fig. 2:**
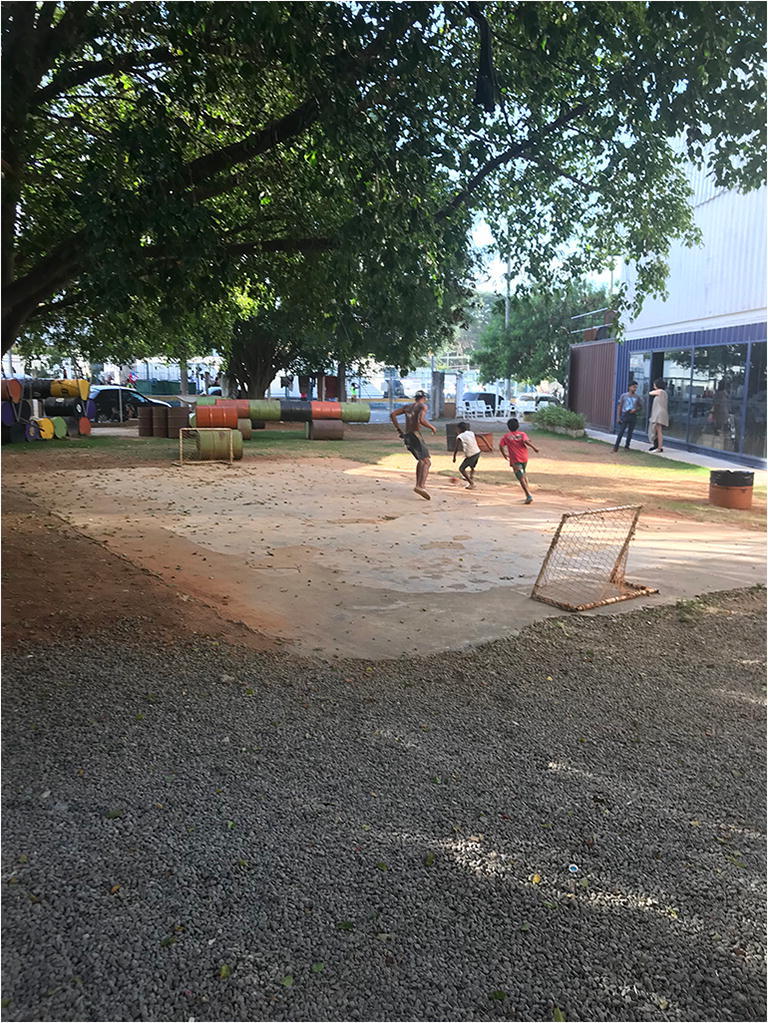
The *Mungunzá Container* Theatre and its playground (Andres, 2018)

In Sao Paulo, the case of the *Mungunzá Container* Theatre (Fig. 2), located in the central neighbourhoods of Luz and Santa Ifigênia, characterised by severe problems with vacant derelict buildings and illegal activities including endemic drug use and trafficking, provides complementary insights on temporary urbanism. The Theatre was developed by a collaboration of artists and designers on a public plot, next to a drug-users medical centre, in collaboration with local authorities. Recycled shipping containers were cut and welded together. While serving a specific cultural need, the site includes a playground, sports court, and gardening installation, which is open to all members of the public during the day [49]. As noted by Rodrigues et al. ([50], p.207), this temporary (which recently became permanent) project has demonstrable benefits, including health benefits: ‘While the surroundings are characterised by a significant number of homeless people sitting and sleeping on the pavement, the site has free access and is used indiscriminately by a different range of people: children playing in the site, homeless people using the sitting area or toilet facilities, cultural users’. This project highlights one of the ways in which temporary urbanism facilitates health outcomes and provides a beacon of hope for the most vulnerable.

#### Everyday Coping and Access to Basic Needs

Temporary and insurgent [27, 28, 51] urbanism, and its socio-economic declinations, contribute to everyday coping including providing access to basic needs. This is the second way temporary urbanisms are connected to health. We have no intention here, as Dovey and King [52] argue, to romanticise those forms of temporary uses led by the most vulnerable. It is however important to reveal the critical roles temporary uses play in supporting (small) health outcomes especially as they tend to be ignored and rejected in policy and urban strategies. Temporary urbanisms are understood in their material forms (i.e. temporary and informal settlements) as providing shelter for the most vulnerable communities and as activities, typically temporary vending stools, offering alternative coping routes through the informal economy and food systems [53, 54]. It also comprises temporary infrastructure arrangements in urban settings allowing off-grid access to water or electricity, or at a micro-level, temporary cooking, or washing facilities. In such configurations, ‘temporary and informal dynamics act as alternative substitutes in places experiencing real difficulties in creating, implementing and enforcing formal planning processes’ ([23], p2). The temporary is one avenue for meeting basic (health) needs.

**Fig. 3:**
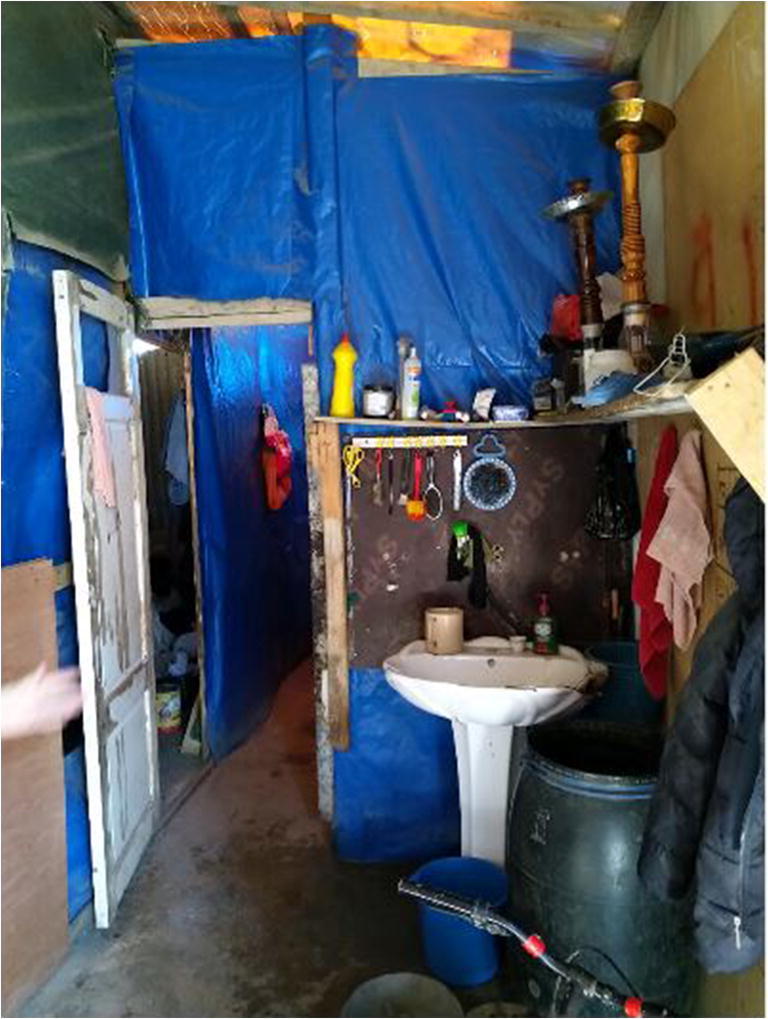
Syrian refugee's tent (Moawad, 2020)

This can be observed in many cities. An excellent example is the Hope for Communities aerial water project in Kibera, Nairobi, Kenya. This is one of largest informal settlements in the world. In 2016, a Kenyan NGO designed an innovative system that provides water from a borehole (deep well) to kiosks using a network of elevated pipes. The elevated system reduced breakage, vandalism, and contamination. The water is treated to make it safer reducing health risks; local residents were employed to sell the water at affordable and stable price ([55]). This clearly is a temporary solution in a temporary setting as a permanent solution is impossible in such settlements. Similar observations can be made in Palestinian camps and Syrian informal tented settlements (ITSs) in Lebanon [29, 56]. Here, as in slums and townships, the connection to health is paradoxical. Refugees’ health vulnerabilities are reinforced by their living conditions which by essence are temporary; ITSs are composed of ‘temporary residential structures, often comprising of plastic-sheeting and timber structures’ ([57], p.118) located on private agricultural lands. These are temporary unregulated structures. ITSs are off-grid with shelters providing limited protection with no adequate hygiene facilitating disease transmission [56]. WASH (water, sanitation, and hygiene) conditions are poor. Controversially, within these temporary and precarious settings, temporary installations ensure daily coping (see Fig. 3), raising hygiene awareness and providing informal and formal education. Temporary and portable schools that cater for ITS’ children have, for example, been provided by local and international NGOs such as the Kayany Foundation and JRS (Jesuit Refugee Service) in the Beqaa area, which hosts most informal ITSs in Lebanon.

#### Crisis, Health, and Temporariness

The third and final way to unwrap the connections between temporary urbanism and health is in relation to the COVID-19 pandemic. This pandemic raises important issues regarding the relationship between health and urban density and challenges existing approaches to working, socialising, and moving around in cities [58]. Urban planning needs to consider the post-pandemic city in the context of immediate responses to COVID-19 combined with temporary urbanism’s role in urban adaptability during times of crisis. There are two distinct contributions: (a) in relation to resilience and the need for adaptable use of buildings and spaces to protect people and address wider concerns about well-being and liveability and (b) in relation to the stigmatisation of everyday coping and informal forms of temporary urbanism driven by health precautionary rationales but with wider heath impacts on the most vulnerable communities. Thus, diligent care is needed. Attempts to manage temporary urbanism, along with controlling temporary practices and uses, can reduce overall community resilience limiting the everyday coping strategies of some of the most vulnerable urban residents.

During the pandemic, lockdowns and social distancing measures impacted on individual behaviours altering mobility patterns in cities. Urban residents across the globe engaged in temporary urbanisms as one coping strategy. Law et al. [59] and Deas et al. [60] and Crump (2020) demonstrated how temporary transformations along with temporary re-arrangements of spaces occurred, enhancing resiliency; this embraces wider ambitions of well-being (especially mental well-being and physical activity). Stadiums, conference centres, and parking spaces were transformed into temporary recovery facilities and hospitals; hotels became quarantine centres or housed the homeless (ibid; [60]). Religious buildings were used as temporary morgues. More importantly, public spaces, parks, and street furniture were radically altered to support social distancing (e.g. wider pavements and footpaths), to maintain economic activities (e.g. restaurant spreading their outdoor dining on pavements), and to accommodate new individual mobilities (temporary cycling lanes, one-way circulation in park, etc.).

Emerging pandemic recovery strategies have focused on meanwhile uses and health. The 2020 London Resilience strategy [21], for example, noted that ‘meanwhile uses have the capacity and flexibility to support, facilitate and implement many of the GLA’s principles of Good Growth by making the best use of land, delivering social outcomes such as neighbourliness and community support, supporting environmental objectives such as improving health and well-being and fostering growth of start-up and scale-up businesses’ (ibid, p.6). Temporary urbanism has been transformed into a solution to address food insecurity, poor health, and well-being, and broader suggestions are being made including developing meanwhile uses for green spaces, allotments to grow organic food, and even temporary health centres, to ‘make streets and neighbourhoods healthier and contribute to Londoners’ well-being’ (ibid, p.35).

In other urban contexts though, the pandemic has led to a stigmatisation of everyday coping strategies and informal forms of temporary urbanism driven by health precautionary measures but with wider heath impacts on the most vulnerable communities. This has been the case in South Africa and Lebanon, for example, where townships and ITSs have been targeted by imposing mobility restrictions, including strict encampments, which not only had direct health implications but also intensified existing vulnerabilities [56]. This has resulted in increased poverty and hunger with wider long-term health implications, for example, on the spread of tuberculosis (TB) [53]. Similar observations regarding health vulnerabilities have been made for migrants and refugees living in ITSs in Lebanon [56, 61]. Here, refugees found themselves in a further limbo state due to strict virus-preventive mechanisms leading to other health consequences, for example, mental health and additional stress due to an increase in isolation and an exacerbated loss of hope noted amongst the youngest generations ([62]). It is, however, noteworthy that preventive health measures (including self-isolation units) relied on temporary re-use and transformations of former ‘buildings’ that are adjacent to ITSs including abandoned structures built by the Lebanese government, non-operational public schools, not in demand suburban hotels, and even non-functional and ill-equipped medical clinics.

### What Are Directions for Health-Led Temporary Urbanism?

Building upon those different features, it is apparent that temporary urbanism and health are strongly connected. Such recognition began to emerge in both research and practice prompted by the 2020/2021 pandemic. It is timely to stretch out the directions for the development of a temporary urbanism-informed approach to health and well-being that engages with research, practice, and policy. This is about developing a health-led approach towards temporary urbanism rather than just considering forms of temporary urbanisms with associated health benefits.

What is apparent from the various types of temporary urbanisms, and how they connect to health (both environmental and public health), is that cities are the primary locations for wealth creation, higher education, urban and national governance, and are key sites for innovation and experimentation; they also contain concentrations of the most vulnerable and disadvantaged. During the COVID-19 crisis, they have also been infection hotspots in which the most vulnerable communities have been the most affected. As such, age, migration and ethnicity, gender, and pre-existing health conditions along with socio-economic backgrounds constitute intersectional layers of disadvantage which have been exacerbated with COVID-19 [63, 64]. Any forms of health-led temporary urbanism must account for the intersectional burdens of the most vulnerable. It is this group which can benefit the most from the relationships between health and different forms of temporary use of urban land and buildings.

Temporary urbanisms provide ways to offer better living conditions—overall well-being—and bring alternatives to foster individual resilience and everyday coping. All are based on adaptable thinking, practices, behaviours, and framework of actions (which can include planning and regulations). This requires new strategic thinking involving appreciating micro-level interventions and needs. Health-led temporary urbanism is a people-centric form of intervention. With COVID-19 rapid improvisations were developed (lockdown, work from home, socially distanced society, and economy) including innovative practices, which would have not been envisaged in other circumstances and which were triggered by ‘experiential learning’. This concept draws upon the literature on organisational adaptation and highlights the importance of understanding the emergence of new routines, or everyday practices, developed inside firms. These routines are path dependent, but new routines will have emerged because of the pandemic displacing existing routines through disruptive innovation [65]. Experiential learning underpins health-led temporary urbanism as a form of place-making that emerges through experimentation and practice. The essence of temporary urbanism relies on the creative and improvised nature of everyday rhythms and practices. In more informal contexts, this resonates with ‘alternative-substitute place-making’ [23] and the contribution this makes to urban transformation and development; it is grounded on processes reflecting a form of ‘permanent impermanence’ (ibid) that highlights inclusion and not exclusion or, in the worst-case scenario, encampments.

Implementing this health-led temporary urbanism agenda requires that this process is situated within a reinterrogation of current models of urban development. The future of the post-pandemic city will include challenging existing models based on urban compactness and enhanced land-use density leading to overcrowded public transport networks. The existing approach is based on design optimisation, the efficient and profitable use of land, and designing out opportunities for the temporary. This does not, as it stands, allow for planning out opportunities for virus transmission and for planning urban environments to maximise health and well-being. The focus must include managing indoor and outdoor people flows reducing transmission opportunities. There are three multi-scalar elements to this challenge which are strongly embedded within the connection between health and temporary urbanism.

First, the built environment, indoors and outdoors, needs to be designed to limit closed social interactions reducing droplet spread during pandemic episode. This includes more holistic and strategic thinking. This involves replacing space optimisation by social distance optimisation, including one-way flows of people between and within buildings combined with effective digital connectivity enabling employees to live away from densely populated urban agglomerations. This is about designing urban environments to facilitate rapid but temporary alterations in the relationship between people and urban space. As such a fourth type of temporary urbanism can be identified with significant policy relevance. It is based on soft and swift transitions for which the mechanism of adaptability and its purpose (e.g. well-being, liveability, community cohesion) are more important than the drivers (e.g. top-bottom or bottom-up or hybrid). This involves rapidly transforming streets into pedestrian spaces to enhance well-being but for a short period, for example, to hold a party or to create a temporary play area.

Second, residential units must be designed as multimodal spaces to support effective work-life balance including dual career homeworking allowing access to green infrastructure to enhance individual and household well-being. Access to green spaces is extremely problematic in very dense areas. A solution may be to turn towards temporary urbanism incorporating movable and temporary gardens and playgrounds. At the neighbourhood and city level, this also includes mapping more exhaustively and making more visible (and accessible) temporary urbanism activities that can underpin health and well-being. This includes enhancing the importance for people to work (and cope) together on projects that enhance social connections while improving social care networks.

Third, a bifold approach to pandemic contingency planning is required. Cities and buildings must be able to respond rapidly to the introduction of effective pandemic control measures while functioning at full capacity during pandemic-free periods. The implication is that all planning applications must come with a virus transmission and management assessment strategy based on temporary adaptations and related management plans to ensure that a building, plot, and area can switch rapidly into pandemic mode. This highlights the importance of this new type of temporary urbanism—soft and swift—and constructed as a response to sudden disruptions requiring prompt adaptation and transformation. It involves a major shift in thinking about the design of the built environment. To achieve this, architectural applications must be changeable and permutable. Spatial functions and land uses are not to be deterministic and limited as both architects, planners, and other built environment experts and clients typically denote them. COVID-19 is a wake-up call across all urban and design disciplines to become more adaptable, incremental, and not presented as a finished product. Flexibility, adaptability, and changeability are pivotal characteristics to induce in those fields of practice and policy when considering future pandemics and human crises.

## Conclusion

This paper has aimed to unwrap the different facets of temporary urbanisms and their connection to health. We are arguing that in the context of the post-pandemic city, there is a need to develop a health-led temporary urbanism agenda constructed upon the different types of temporary urbanism and applicable in various settings both in the Global South and North. Such approach contributes to the call for more trans- and inter-disciplinary discussions allowing to more thoroughly link urban planning and development with health.

It is apparent that health-led temporary urbanism must be supported by agent-based modelling to test designs and to intelligently simulate people flows and encounters. The emphasis must be on designing healthy cities and buildings in an adaptable, sustainable, and resilient way. This requires developing a new balance between health and urban planning including designing multifunctional spaces into urban environments that provide sites for temporary urbanism-related activities. A healthy city is an adaptable city and one that provides opportunities for citizen-led interventions intended to enhance well-being by blending the temporary with the permanent and the planned with the improvised.

## References

[1] Hall P (2014). Cities of tomorrow. An intellectual history of urban planning and design since 1888 (4th edition).

[2] Lowe M, Hooper P, Jordan H, Bowen K, Butterworth I, Giles-Corti B. Evidence-Informed Planning for Healthy Liveable Cities: How Can Policy Frameworks Be Used to Strengthen Research Translation? Curr Environ Health Rep. 2019;6(3):127–136. 10.1007/s40572-019-00236-6 31134515

[3] • Pineo H. Towards healthy urbanism: inclusive, equitable and sustainable (THRIVES) – an urban design and planning framework from theory to praxis. Cities & Health. 2020. 10.1080/23748834.2020.1769527**Latest paper discussion the connection between health and urban planning**. 10.1080/23748834.2020.1769527 PMC7616730 39444987

[4] Rydin Y (2010). Governing for sustainable urban development.

[5] Marsh, R., Pilkington P., Marco E., Rice L. Evaluating a workforce development programme: bringing public health into architecture education in England. Cities & Health 2020: 10.1080/23748834.2020.1736738

[6] Pilkington P, Marco E, Grant M, Orme J (2013). Engaging a wider public health workforce for the future: a public health practitioner in residence approach. Public health..

[7] CSDH (2008). Closing the gap in a generation: health equity through action on the social determinants of health. Final report of the commission on social determinants of health.

[8] Gelormino E, Melis G, Marietta C, Costa G (2015). From built environment to health inequalities: an explanatory framework based on evidence. Preventive medicine reports..

[9] Northridge ME, Freeman L (2011). Urban planning and health equity. Journal of urban health..

[10] Andres L, Zhang Y (2020). Transforming cities through temporary urbanism - a comparative overview.

[11] • Andres, L, Kraftl, L. New directions in the theorisation of temporary urbanisms: adaptability, activation and trajectory. Progress in Human Geography, 2021: 10.1177/0309132520985321. **Latest reference linking adaptability and temporary urbanism**

[12] Mackenzie D (2020). COVID-19. The pandemic that never should have happened and how to stop the next one.

[13] Adams D, De Sousa C, Tiesdell S (2010). Brownfield development: a comparison of North American and British approaches. Urban Studies.

[14] Andres L (2012). Levels of governance and multi-stage policy process of brownfield regeneration: a comparison of France and Switzerland. International Planning Studies.

[15] Iveson K (2013). Cities within the city: do-it-yourself urbanism and the right to the city. International Journal of Urban and Regional Research..

[16] Mould O (2014). Tactical urbanism: the new vernacular of the creative city. Geography Compass..

[17] Merker B, Hou J (2010). Taking place: Rebar’s absurd tactics in generous urbanism. Insurgent Public Space: Guerrilla Urbanism and the Remaking of Contemporary Cities.

[18] Groth J, Corijn E (2005). Reclaiming urbanity: indeterminate spaces, informal actors and urban agenda setting. Urban Studies..

[19] Andres L (2013). Differential spaces, power-hierarchy and collaborative planning: a critique of the role of temporary users in shaping and making places. Urban Studies.

[20] Harris E (2015). Navigating pop-up geographies: urban space–times of flexibility, interstitially and immersion. Geography Compass.

[21] Major of London. London City Resilience Strategy, https://www.london.gov.uk/what-we-do/fire-and-resilience/london-city-resilience-strategy. London. Accessed on 03 January 2021

[22] Madanipour A (2017). Cities in time: temporary urbanism and the future of the city.

[23] Andres L, Bakare, H, Bryson, JR, Khaemba, W, Melgaco, L, MwanikI, G. Planning, temporary urbanism and citizen-led alternative-substitute place-making in the Global South, Regional Studies. 2019: 10.1080/00343404.2019.1665645

[24] Bryson, J. R. Obsolescence and the Process of Creative Reconstruction. Urban Studies, 1997;34(9):1439–1458.

[25] Andres, L, La ville face aux incessants changements de ses formes et de ses fonctions : la mutabilité comme constitutive du fait urbain in ROSBOCH M., BERTRAND G. (Eds.), Le dinamiche del cambiamento. Cultura, cittadinanza, economia nelle regioni alpine occidentali tra età moderna e globalizzazione, Libreria Stampatori, Turin, 2009;51–66.

[26] Lerup L (1977). Building the unfinished: architecture and human action.

[27] Miraftab F. Insurgency, planning and the prospect of a humane urbanism. Keynote Speech. World Congress of Planning Schools “Global Crisis, Planning and Challenges to Spatial Justice.” 3-7 July 2016, Rio de Janeiro. Available from: https://www.academia.edu/5009516/Displacement_Framing_the_global_relationally. Accessed on 05 April 2020

[28] Miraftab F, Gunder M, Madanipour A, Watson V (2017). Insurgent practices and decolonization of future(s). The Routledge handbook of planning theory.

[29] Moawad P, Andres L, Zhang Y (2020). Temporary forms of urbanism in contested urban spaces in Lebanon: the case of Dbayeh camp. Transforming Cities Through Temporary Urbanism. A Comparative Overview.

[30] Bishop P, Williams L (2012). The temporary city.

[31] Bishop P (2015). From the subversive to the serious. Counterpoint.

[32] Bishop P, Roggema R (2019). Urban design in the fragmented city. Contemporary urban design thinking: the Australian approach.

[33] Oswalt P, Overmeyer K, Misselwitz P (2017). The power of temporary.

[34] Pinard J, Andres L, Zhang Y (2020). Developing ‘transient urbanism’ as a new urban and real estate strategy: the case of the French National Railway Company (SNCF). Transforming Cities Through Temporary Urbanism. A Comparative Overview.

[35] Maeder T, Andres L, Zhang Y (2020). Artistic events as planning practice: hybridisation, expectations and pitfalls in three Swiss case studies. Transforming Cities Through Temporary Urbanism. A Comparative Overview.

[36] Zhang AY, Andres L, Zhang Y (2020). Address urban regeneration challenge with temporary creative uses: the case of Beijing’s Dashilar Area. Transforming Cities Through Temporary Urbanism. A Comparative Overview.

[37] Crump, L. Meanwhile uses in the city – should this be the new normal? https://blogs.lse.ac.uk/progressingplanning/2020/07/06/meanwhile-uses-in-the-city-should-this-be-the-new-normal/. Accessed on 15 November 2020a

[38] Crump L, Andres L, Zhang Y (2020). Reimagining urban planning: from institution to innovation. A comparative exploration of temporary urbanism and the future of city marking. Transforming Cities Through Temporary Urbanism. A Comparative Overview.

[39] Garcia M, Andres L, Zhang Y (2020). The usefulness of temporary use: narratives from Santiago’s contemporary urban practices. Transforming Cities Through Temporary Urbanism. A Comparative Overview.

[40] Pike, A., Dawley, S., Tomaney, J. Resilience, Adaptation and Adaptability (March 2010). Cambridge Journal of Regions, Economy and Society, 2010;3(1):59-70.

[41] Tonkiss F (2013). Austerity urbanism and the makeshift city. City..

[42] Wilkinson RG, Marmot M (2003). Social determinants of health: the solid facts.

[43] Kestens Y, Winters M, Fuller D, Bell S, Berscheid J, Brondeel R, et al. INTERACT: a comprehensive approach to assess urban form interventions through natural experiments. BMC Public Health. 2019;51:51. 10.1186/s12889-018-6339-z. 10.1186/s12889-018-6339-z PMC6327503 30630441

[44] Patton GC, Sawyer SM, Santelli JS, Ross DA, Afifi R, Allen NB, et al. Our future: a Lancet commission on adolescent health and wellbeing. Lancet. 2016;387(10036):2423–78. 10.1016/S0140-6736(16)00579-1 PMC5832967 27174304

[45] DCLG (2009). Looking after our town centres.

[46] Assembly L (2013). Open for business: empty shops on London’s high streets.

[47] Meanwhile Foundation. The Meanwhile Foundation is Open for Business, 2016. Available from: http://www.meanwhile.org.uk/, 6.06.2016. Assessed on 6 June 2016.

[48] https://loughboroughjunction.org. Accessed 3 Jan 2021.

[49] Companhia Mungunza de Teatro. Teatro de Container Mungunza. http://www.ciamungunza.com.br/en/conteiners. Accessed 04 March 2018

[50] Rodrigues L, Soares Goncalves L, Tubelo R, Porter N, Mirzaei P, Kraftl P, et al. Temporary urban interventions in the Luz and Santa Ifigênia neighbourhoods in São Paulo, Brazil. In: Andres L, Zhang Y, editors. Transforming Cities Through Temporary Urbanism - A Comparative Overview. Cham: Springer; 2020. p. 199–214.

[51] Miraftab F (2009). Insurgent planning: situating radical planning in the global south. Planning Theory..

[52] Dovey K, King R (2012). Informal urbanism and the taste for slums. Tourism Geographies..

[53] Denoon-Stevens S, du Toit K, Bryson JR, Andres L, Ersoy A, Reardon L (2021). The job-food-health nexus in South African townships and the impact of COVID-19. Living with pandemics: places, people, policy and rapid mitigation and adaptation to Covid-19.

[54] Skinner, C, Watson, V. Viewpoint: planning and informal food traders under COVID-19: the South African case. Town Planning Review. 2020: 10.3828/tpr.2020.38.

[55] Global Waters. “The rise of SkyWater” – Challenges of an aerial water distribution system, Available from https://www.globalwaters.org/us-global-water-strategy-stories/rise-skywater-challenges-aerial-water-distribution-system. Accessed 17 Feb 2021.

[56] Moawad P, Andres L (2020). Tackling COVID-19 in informal tented settlements (Lebanon): an assessment of preparedness and response plans and their impact on the health vulnerabilities of Syrian refugees. Journal of Migration and Health..

[57] Sanyal R (2017). A no-camp policy: interrogating informal settlements in Lebanon. GeoForum..

[58] Nieuwenhuijsen, M. Post-COVID-19 cities: new urban models to make cities healthier. 2020. Available from: https://www.isglobal.org/en/healthisglobal/-/custom-blog-portlet/post-covid-19-cities-new-urban-models-to-make-cities-healthier/4735173/0. Assessed on 15 January 2021

[59] Law L, Azzali S, Conejos S (2020). Planning for the temporary: temporary urbanism and public space in a time of COVID-19. Town Planning Review..

[60] Deas I, Martin M, Hincks S (2020). Temporary urban uses in response to COVID-19: bolstering resilience via short-term experimental solutions. Town Planning Review..

[61] UNHCR. Progress Report - 3RP Regional Refugee & Resilience Plan in Response to the Syria Crisis. Available from: https://reliefweb.int/report/syrian-arab-republic/2020-progress-report-3rp-regional-refugee-resilience-plan-response-syria. 2020. Accessed on 20 November 2020

[62] Danish Refugee Council. The impact of Covid-19 on Syrian refugee adolescent wellbeing and coping in Lebanon, DRC, Copenhagen. 2020.

[63] Hankivsky, O, Kapilashrami, A. Beyond sex and gender analysis: an intersectional view of the COVID-19 pandemic outbreak and response. Available from : https://www.qmul.ac.uk/gpi/media/global-policy-institute/Policy-brief-COVID-19-and-intersectionality.pdf Assessed on 15 January 2021.

[64] Ho EL, Maddrell A (2021). Intolerable intersectional burdens: a COVID-19 research agenda for social and cultural geographies. Social & Cultural Geography..

[65] Bryson JR, Vanchan V, Kalafsky RV, Bryson JR, Kalafsky RV, Vanchan V (2021). Reframing urban theory: smaller towns and cities, forms of life, embedded plasticity, and variegated urbanism. Ordinary cities, extraordinary geographies: people, place and space.

